# Unbiased Method to Determine Articular Cartilage Thickness Using a Three-Dimensional Model Derived from Laser Scanning: Demonstration on the Distal Femur

**DOI:** 10.3390/bioengineering11111118

**Published:** 2024-11-06

**Authors:** Valentina Campanelli, Maury L. Hull

**Affiliations:** 1 THINK Surgical, Fremont, CA 94538, USA; vcampanelli@thinksurgical.com; 2 Department of Biomedical Engineering, University of California Davis, Davis, CA 95616, USA; 3 Department of Mechanical Engineering, University of California Davis, Davis, CA 95616, USA; 4 Department of Orthopaedic Surgery, University of California Davis Medical Center, Sacramento, CA 91817, USA

**Keywords:** articular cartilage, laser scanner, computed tomography, fiducial markers, dermestid beetles

## Abstract

Measuring articular cartilage thickness from 3D models developed from laser scans has the potential to offer high accuracy. However, this potential has not been fulfilled, since generating these models requires that the cartilage be removed, and previous methods of removal have led to systematic errors (i.e., bias) due to changes in the overall dimensions of the underlying bone. The objectives were to present a new method for removing articular cartilage, quantify the bias error, and demonstrate the method on the distal (i.e., 0° flexion) and posterior (i.e., 90° flexion) articular surfaces of example human femurs. The method consisted of creating a 3D articular cartilage model from high-accuracy (i.e., precision = 0.087 mm) laser scans before and after cartilage removal using dermestid beetles to remove the cartilage. Fiducial markers were used to minimize errors in registering surfaces generated from the two laser scans. To demonstrate the method, the cartilage thickness was computed in distal and posterior subregions of each femoral condyle for three example cadaveric specimens. The use of dermestid beetles did not introduce measurable bias, and the previously reported precision achieved in 3D cartilage models with the laser scanner was 0.13 mm. For the different subregions, the cartilage thickness ranged from 1.5 mm to 2.0 mm. A method of imaging by means of laser scanning, cartilage removal by means of dermestid beetles, and 3D model registration by means of fiducial markers ensured that cartilage thickness on the articular surface of the long bones of the knee was determined with negligible bias and a precision of 0.13 mm. With this method, the potential to measure cartilage thickness with high accuracy based on 3D models developed from laser scans can be fully realized.

## 1. Introduction

Determining cartilage thickness on the articular surfaces of the long bones of the human knee is important in a number of applications. Applications include characterizing the morphology of articular surfaces [1,2], incorporating articular cartilage into computational models [3,4], studying knee kinematics [5,6,7,8,9], optimizing surgical techniques for total joint arthroplasty that require that the thickness of worn articular cartilage be determined [10,11], and tracking the progression of degenerative changes [12,13].

Various methods have been used to measure articular cartilage thickness, including both non-invasive and invasive methods. Non-invasive methods include three-dimensional (3D) models of articular cartilage derived from magnetic resonance (MR) images (MRI) [14,15,16,17,18], computed tomography (CT) arthrograms [19], and ultrasound (US) [20,21]. Invasive methods include stereophotogrammetry (SPG) [18,22], laser scanning [14,23,24,25], sectioning and direct measurement [21,26], and needle probe [26]. 

Arguably measuring cartilage thickness based on 3D models derived from laser scans offers potential for the highest accuracy in relation to the other methods mentioned above [24,25]. Depending on the scanner, laser scanning has a documented precision in scanning the distal femoral articular surface ranging from 67 to 92 microns [27]. Since two scans are required (i.e., one before and one after cartilage removal), the precision in measuring cartilage thickness becomes σ_t_ = sqrt (σ^2^ + σ^2^) where σ is the precision of an individual scan. Hence, the precision in cartilage thickness ranges from 94 to 130 microns. Even at 130 microns, the precision is only about 6% of the mean 2 mm articular cartilage thickness on the distal human femur, in which case the random error is relatively small.

Although the random error has been quantified, to fully characterize accuracy, the systematic error or bias must be quantified as well [28]. Previous methods of removing articular cartilage affected the dimensions of the underlying bone, thus introducing bias error. Removal techniques used previously involved maceration and included immersion in sodium hypochlorite (bleach) [14,23,24,25], acetone [29], or boiling in water [29,30]. Treatment by chemicals causes damage to the cortical tissue due to the corrosive action on calcium [31], and boiling also causes damage to the cortical bone [32]. As a result, the overall dimensions of the bone decrease by up to 0.2–0.5 mm [29,30]. With a 2 mm cartilage thickness, these effects translate into large relative bias errors, up to 25%. Hence, a method for removing articular cartilage with demonstrated minimal bias is needed for 3D models developed from laser scans to realize the potential for high accuracy in measuring cartilage thickness.

An untested method, which involves no chemicals or high-temperature immersion and may better preserve the overall bone dimensions, thus minimizing bias, is to use dermestid beetles to remove the articular cartilage by ingestion [33,34]. However, no study known to the authors has used this removal technique in the development of 3D cartilage models to determine cartilage thickness.

The present study had three objectives. The first was to present a new method to determine cartilage thickness on the long bones of the knee by using laser scanning in conjunction with dermestid beetles to remove the articular cartilage, a second was to determine whether the use of dermestid beetles to remove the articular cartilage preserves the morphology (i.e., size) of the bone, thus minimizing bias, and the third was to demonstrate the method by determining the cartilage thickness on the distal and posterior surfaces of several cadaveric human femurs. A method of cartilage removal with minimal bias and a small random error of 6%, as noted above, would fully realize the potential of measuring cartilage thickness based on 3D models developed from laser scans with high accuracy. 

## 2. Methods

**Figure 1:**
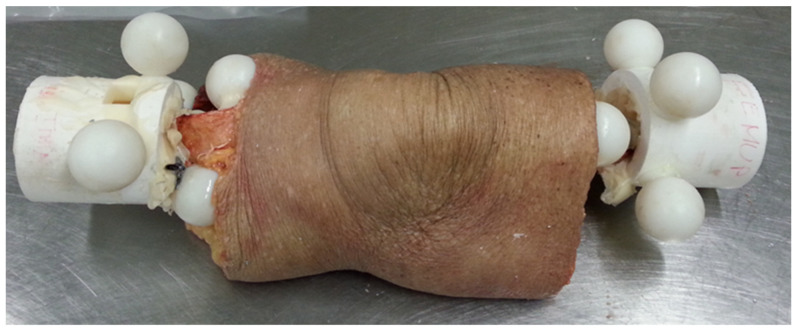
Knee specimen after potting and attaching the fiducial markers. Four or five markers were attached to the potting cup using a nylon threaded stud and two-part resin epoxy glue. Two markers were attached directly to the bone using nylon threaded studs and methylmethacrylate.

To demonstrate the method, three unpaired fresh-frozen human cadaveric knee specimens (average age ≈ 81years) free from degenerative joint disease were included. After transecting specimens at the mid-points of the long bones to isolate the knee, the proximal section of the femur and the distal section of the tibia were potted using bone cement (Figure 1).

**Figure 2:**
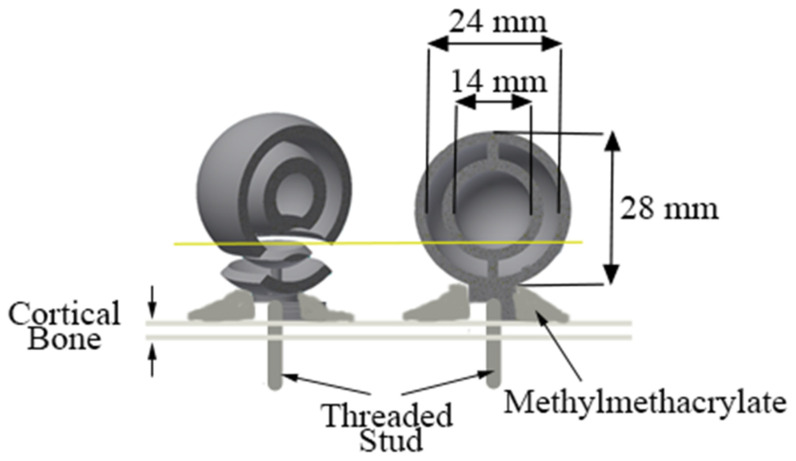
Diagram of the fiducial marker design, which included inner and outer spheres of 14 mm and 24 mm in diameter, respectively. The inner sphere diameter was such that it could be visualized and segmented in at least 11 CT slices at 1.25 mm thickness (22 slices for a slice thickness of 0.625 mm). The outer sphere diameter was such that it could be visualized and segmented into at least 12 MRI slices at 2 mm slice thickness. A threaded nylon stud connected the marker to the bone through methylmethacrylate or to the potting cup through epoxy.

Fiducial markers were fabricated using a 3D printer (Objet Connex 260V, Stratasys, Proto3000, Vaughan, ON, Canada) as semi-hollow spheres of 28 mm in diameter (Figure 2). Six to seven fiducial markers were attached to each femur and tibia. Four to five markers were attached to the potting cup, while the remaining two markers were attached directly to the bone (Figure 1).

Before and after cartilage removal, CT scans and laser scans were performed, and 3D models were created from each of the scans. The articular cartilage thickness was determined from the 3D models created from the laser scans. The 3D models created from the CT scans were for different purposes. Before cartilage removal, one purpose was to enable the orientation of the knee into standard planes, and another purpose was to provide a baseline morphology for the bones. After cartilage removal, the purpose was to assess whether the cartilage removal process affected bone morphology.

A 32-slice CT scanner (GE LightSpeed, Chicago, IL, USA) was used for the CT scans. The CT imaging protocol included a slice thickness of 0.625 mm, 120 kVp, smart mA, no slice gap/overlap, a 512 × 512 image matrix, and a pixel size of 0.39 mm. The CT images of each knee specimen and the fiducial markers were segmented using the automatic tools in Mimics^®^ (Materialise, Leuven, Belgium) and refined manually. Using Mimics, 3D models of the intact extended knee consisting of the femur, tibia, and fiducial markers were constructed using a variation of the classic “marching cubes algorithm”. The settings for 3D model construction were the following: interpolation method “gray value”, preferred “accuracy”, shell reduction to 1, no matrix reduction applied, and a smoothing factor of 0.5 using 7 iterations.

After the first of the two CT scans was performed, soft tissues were removed and the joint was disarticulated. Using a high-accuracy laser scanner with a point-to-point resolution of 0.050 mm, the femur and tibia were scanned to generate 3D point clouds, which included articular cartilage (Metrascan 3D, Creaform, Levis, QC, Canada). Following the laser scan, the articular cartilage and remaining soft tissues were removed using dermestid beetles [33,34]. The removal of articular cartilage via dermestid beetles lasted, on average, a period of 4 weeks for each bone, during which time the bones were water-sprayed every 2–3 days, covered to avoid desiccation, and maintained at room temperature. To generate 3D point clouds without articular cartilage, the femur and tibia were laser-scanned a second time. From each 3D point cloud, 3D models of the femur and tibia were created using Metrascan software. The repeatability error (root mean square deviation) in scanning a distal femur was 87 microns [27]. Three individual bones without cartilage were CT-scanned a second time to assess any change in bone morphology caused by the cartilage removal process, and 3D models were created as described above.

**Figure 3:**
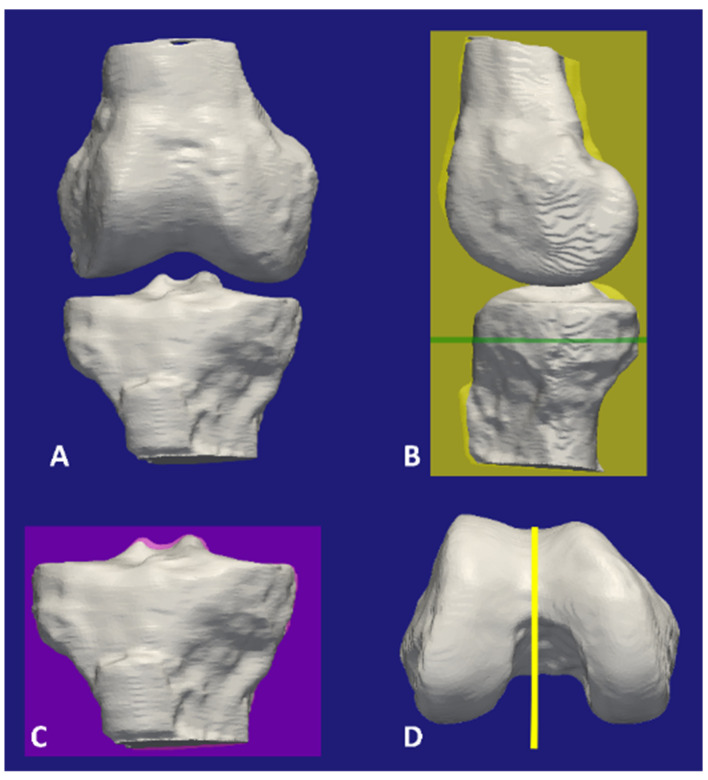
Images showing a 3D model of an extended right knee (**A**) and the steps for orientating the knee in the standard planes (**B**–**D**). The knee model was developed by registering the 3D models of the tibia and femur generated from laser scanning with the respective bones in the 3D model of the intact knee from the first CT scan (**A**). The standard sagittal plane (yellow) was formed by superimposing the posterior femoral condyles [5,6,7,8,9], the standard axial plane (green) was normal to the standard sagittal plane and parallel to the medial surface of the tibial plateau (**B**), and the standard coronal plane (purple) was mutually perpendicular to the other two standard planes (**C**). The line on the distal femur was parallel to the standard sagittal plane (**D**).

To reassemble the individual 3D models of the tibia and femur developed from laser scanning into a 3D laser-scanned extended knee model, the laser-scanned models of the femur and tibia with and without articular cartilage were registered using fiducial markers. Next, the individual 3D models of the tibia and femur developed from laser scanning without articular cartilage were registered to the respective bones in the CT model of the intact extended knee. The transformations used to register the laser-scanned models of the femur and tibia without articular cartilage were applied to the laser-scanned models of the femur and tibia with articular cartilage to superimpose the respective 3D models spatially in a consistent position and orientation. The 3D models were registered using the centers of spheres that were generated through a best fit of the 3D models of the fiducial markers using a least-squares fitting method implemented in Geomagic (Geomagic^®^, 3D Systems, Cary, NC, USA). Following the registration process, the extended 3D laser-scanned knee model, which included the articular cartilage, was aligned in standard planes (Figure 3).

To assess whether bone morphology changed during cartilage removal, morphological differences in the three 3D bone models generated from the CT scans were determined by computing the root mean squared deviation (RMSD) and average deviation (AD). To ensure that any morphological differences found in the CT models before and after cartilage removal were not due to the repeatability error in the segmentation, the repeatability error in the segmentation of the CT images was computed. The same set of CT images was segmented five times to create five 3D models, which were compared pairwise (i.e., ten comparisons). From these ten comparisons, the mean RMSD and AD values were computed. If deviations due to the repeatability error in segmentation were the same as deviations due to apparent morphological changes after cartilage removal, then it could be concluded that the method of cartilage removal did not introduce appreciable changes in bone morphology (i.e., bias error was negligble).

**Figure 4:**
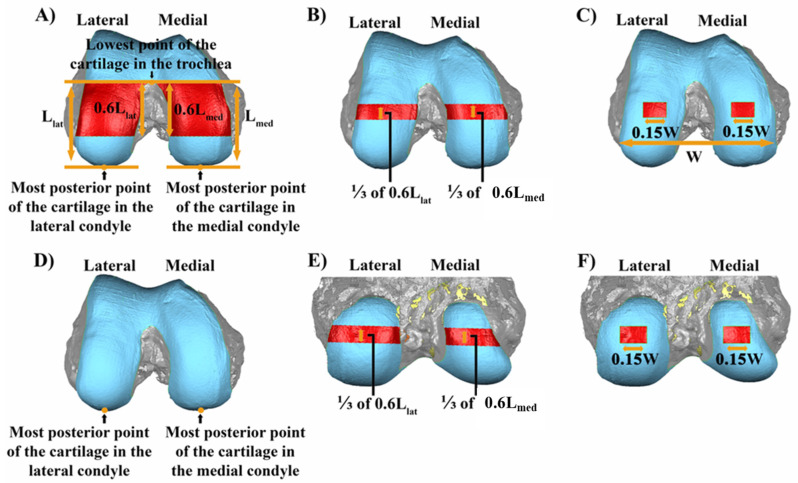
Composite showing how the subregions in the femoral condyles at 0° (**A**–**C**) and 90° (**D**–**F**) were defined. To define the subregions at 0°, the weight-bearing region of the femur was selected. The weight-bearing region was bounded anteriorly by the medio-lateral line intersecting the lowest point of the articular cartilage in the trochlea and extending 60% of the distance to the most posterior point of the articular cartilage (**L_lat_** and **L_med_** for the lateral and medial condyles, respectively) (**A**). In the anterior–posterior direction, the central 1/3 portion of the weight-bearing region was selected on each femoral condyle (**B**). The medial–lateral width of the selected 1/3 portion of the weight-bearing region on each femoral condyle was reduced to 15% of the total medial–lateral width **W** of the articular cartilage and centered at the centroid of the central 1/3 portion of the weight-bearing region. The total medial–lateral width **W** of the articular cartilage was defined as the distance between the most medial point and most lateral point of the articular cartilage (**C**). To define the subregions at 90°, the most posterior point of the articular cartilage was selected one each femoral condyle (**D**). In the proximal–distal direction, the region centered about the most posterior point of each femoral condyle with the same length as the subregion at 0° of flexion was selected (**E**). The medial–lateral width of the selected central portion was reduced to 15% of the total medial–lateral width of the articular cartilage and centered at the centroid of the previously selected central portion (**F**).

As an example of cartilage thickness measurement using the 3D models, subregions on the medial and lateral femoral condyles were defined at 0° and 90° flexion, as described in the caption to Figure 4, to determine the articular cartilage thickness. Once the subregions were defined, the articular cartilage thickness at each point in a subregion was calculated using the closest point algorithm implemented in MATLAB^®^ (Mathworks, Natick, MA, USA). The average and standard deviation of thickness over all points in the subregion was calculated for each femur.

## 3. Results

Mean deviations in morphology of the CT bone models generated before and after cartilage removal were an RMSD of 0.3 mm and AD of 0.1 mm. These deviations were the same as those from the pairwise comparisons of the five 3D bone models generated based on repeated segmentation. Hence, any change in bone morphology manifesting as bias error caused by using dermestid beetles for cartilage removal was not quantifiable.

For the different subregions of the medial and lateral femoral condyles, example measurements of cartilage thickness yielded averages that ranged widely. The thinnest was 1.2 mm on the medial femoral condyle at 90° flexion for Specimen 3, and the thickest was 2.8 mm on the lateral femoral condyle at 0° flexion for the same specimen (Table 1). Standard deviations ranged from 0.1 mm to 0.4 mm. The mean average (i.e., over all specimens and subregions) was 1.9 mm.

## 4. Discussion

Three key aspects of the method of determining articular cartilage thickness with high accuracy from 3D models developed from laser scans were (1) the use of a laser scanner with a precision of 0.087 mm to determine 3D models before and after cartilage removal, (2) the use of dermestid beetles to remove the articular cartilage while preserving the morphology of the distal femur, and (3) the use of fiducial markers to register bone models. Each of these aspects will be discussed in turn.

Using the Metrascan laser scanner offered advantages that ensured the generation of highly accurate 3D models. The Metrascan has a documented repeatability (i.e., precision) of 87 microns based the comparison of 3D bone models of the distal femur created from multiple scans [27]. Another advantage of the Metrascan is that it enabled the femur and tibia to be scanned only once with cartilage and once without cartilage, without the need to perform multiple scans and register the scans in post-processing to obtain the entire 3D surface [24,25]. Since the cartilage thickness was derived from two scans, the overall point-wise precision for the cartilage model per se is 1.414 (i.e., square root of 2) × 0.087 mm ≈ 0.13 mm.

There was no measurable change in bone morphology using dermestid beetles to remove the articular cartilage as opposed to other methods, such as using a chemical solution that causes measurable changes in bone morphology [29,30]. The advantage in maintaining the bone morphology was that the bias error was negligible, in which case the only quantifiable error was precision, as noted above. Accordingly, the use of dermestid beetles to remove articular cartilage is a viable option for reaching the full potential of 3D models derived from laser scans to determine cartilage thickness with high accuracy.

To register bone models, fiducial markers were mounted on the bone and registered, rather than shape-matching the models using, for example, a method such as the iterative closest point method. Registering the fiducial markers rather than shape-matching was preferred to maintain the bone models in a consistent coordinate system. If the coordinate systems had differed, then this difference would have introduced an error to the determination of the cartilage thickness using a closest point algorithm.

In comparing our results with those from the literature, comparison will be limited to those studies where the articular cartilage was removed and accuracy metrics were reported, since these comparisons are the most relevant. An early study used stereophotogrammetry to determine cartilage thickness on the articular surfaces of the patella, tibia, and femur [22]. Articular cartilage was removed using bleach, which reduces the overall dimensions of the bone, as noted in the Introduction [29,30]. The reported precision was 0.13 mm, which compares favorably with the precision obtained for the laser scanner used herein. However, the bias error due to decreases in the overall bone dimensions was not reported.

A later study using a laser scanner also removed articular cartilage using bleach and compared the volume of cartilage based on the 3D model with the volume removed measured using water immersion [25]. Although a difference of 4% was reported, it is unclear how this difference translates to a difference in thickness. Also, both methods were prone to the same bias error, since both methods would have been affected by decreases in overall bone dimensions. In a subsequent study, the same authors assessed repeatability by scanning the same femur multiple times with and without articular cartilage [24]. Using the same methods as in the earlier study, a coefficient of variation (CV) of 5.3% was reported. Using new methods to address the sensing (i.e., depth) and sampling (i.e., spatial resolution) errors, the CV was reduced to 1.4%. It is important to note that the CVs are based on standard deviations computed after low-pass filtering. Accordingly, the CVs indicate region-wise rather than point-wise precision. Hence, the point-wise precision could not be determined, and bias error was not reported.

As the forgoing indicates, the literature that has evaluated errors in cartilage thickness based on 3D models generated from laser scans is scarce. Hence, by fully quantifying errors according to standard methods [28], our study fills an important gap in the literature.

One perceived limitation of our method might be the use of dermestid beetles to remove the articular cartilage because of the time required. However, for cartilage thickness to be measured from 3D models developed from laser scans, cartilage must be removed by some method, in which case time will be taken for removal. If the goal is to accurately determine cartilage thickness, then the advantage of negligible bias error outweighs the disadvantage of the additional time required for cartilage removal using dermestid beetles. Although uncommon in orthopedic biomechanics research, and hence relatively unknown, colonies of dermestid beetles are readily available from multiple sources including Amazon.

Finally, our study did not compare cartilage thickness to other methods. Any meaningful comparison would have required comparison with another method requiring cartilage removal where bias could be introduced by the removal process. Although this was not possible because of the destructive nature of articular cartilage removal, it would be possible to use the methods described herein with another method of cartilage removal to quantify the bias error associated with that method.

## 5. Conclusions

Our study used methods of imaging by means of laser scanning, cartilage removal by means of dermestid beetles, and 3D model registration by means of fiducial markers, which realized the potential in using 3D models developed from laser scans to measure cartilage thickness with high accuracy. Using a laser scanner with a precision of 0.087 mm, this method determines cartilage thickness with negligible bias and with a precision of 0.13 mm, which translates to a 6% relative error when determining cartilage thickness on the distal human femur. Accordingly, we expect this method to be applied in studies that demand the highest possible accuracy.

## Figures and Tables

**Table 1:** Average and standard deviation values in mm for cartilage thickness in each subregion for each specimen tested. The overall mean average (i.e., mean of mean averages) was 1.9 mm.

	0° of Flexion		90° of Flexion	
Specimen	Medial	Lateral	Medial	Lateral
Specimen 1	2.0 (0.4)	2.0 (0.3)	1.9 (0.3)	2.0 (0.1)
Specimen 2	2.2 (0.3)	1.5 (0.3)	1.6 (0.1)	1.9 (0.1)
Specimen 3	1.5 (0.4)	2.8 (0.2)	1.2 (0.3)	2.3 (0.3)
Mean Average	1.9	2.1	1.6	2.1

## Data Availability

Full results are provided in the manuscript.
